# Tomographic Evaluation of Alveolar Ridge Preservation With a Porcine Pericardium Collagen Membrane and Xenograft: A Case Report

**DOI:** 10.7759/cureus.106030

**Published:** 2026-03-28

**Authors:** María José Jarrín Peñafiel, Pedro Alberto López Reynoso, Ana Gabriela Sifuentes Carrillo, Christian Fernando Arciniegas Parra, Beatriz Raquel Yáñez Ocampo, Daniela Carmona Ruiz, César Esquivel Chirino

**Affiliations:** 1 Specialization in Periodontics and Implantology, Division of Postgraduate Studies and Research, Faculty of Dentistry, National Autonomous University of Mexico, Mexico City, MEX; 2 Advanced Specialization in Oral, Surgical and Prosthetic Implantology, Division of Postgraduate Studies and Research, Faculty of Dentistry, National Autonomous University of Mexico, Mexico City, MEX; 3 Specialization in Oral Prosthetics and Implantology, Division of Postgraduate Studies and Research, Faculty of Dentistry, National Autonomous University of Mexico, Mexico City, MEX; 4 Department of Orthodontics, Faculty of Dentistry, National Autonomous University of Mexico, Mexico City, MEX; 5 Area of Basic Medical Sciences, Faculty of Dentistry, National Autonomous University of Mexico, Mexico City, MEX

**Keywords:** alveolar ridge preservation, bone graft collagen membrane, cone-beam computed tomography (cbct), dental implants, regenerative membrane

## Abstract

Bone resorption after tooth extraction can compromise alveolar ridge volume and limit implant-supported rehabilitation. Alveolar ridge preservation (ARP) using xenografts and resorbable collagen membranes has been proposed to reduce post-extraction dimensional changes; however, outcomes may vary depending on site characteristics and clinical conditions. The objective of this clinical case was to evaluate multidimensional ridge changes following alveolar preservation using a porcine pericardium collagen membrane and bovine bone xenograft prior to implant placement, assessed by cone beam computed tomography (CBCT). A 61-year-old male patient with Stage IV periodontitis and severe mobility underwent multiple extractions followed by alveolar preservation. CBCT analysis was performed at predefined extraction sites in the maxilla (#11, #12, #13, #21, #22, and #23) and mandible (#33, #34, #43, and #44). The measurement levels used were: coronal width (CW), the horizontal distance between the buccal and palatal/lingual bone crests at the coronal level; apical width (AW), the horizontal distance at the apical level; buccal apicoronal height (BH/AC), the distance from the apex to the buccal crest; and palatal/lingual apicoronal height (PLH/PAC-LAC), the distance from the apex to the palatal or lingual crest. CBCT scans were obtained under standardized acquisition parameters and recorded using voxel-based rigid alignment across sagittal slices. At 12 months, ridge dimensions remained stable within sub-millimeter ranges. In the maxilla, coronal width changed from 8.2 to 8.4 mm and apical width from 8.8 to 9.9 mm. In the mandible, coronal width changed from 9.2 to 9.6 mm and apical width from 9.9 to 10.1 mm. Vertical dimensional changes (buccal and palatal/lingual) were less than 1 mm. Eight implants were placed without additional augmentation procedures, and a control CBCT performed three months later confirmed dimensional stability at implant sites. In this patient, the combined regenerative approach was associated with favorable ridge stability prior to implant placement. Further studies are required to confirm the predictability of this approach.

## Introduction

The main function of the dental alveolus is to support dental roots [1]. After tooth extraction, the socket undergoes a healing and remodeling process characterized by the formation of a clot, an inflammatory phase, and early osteoclastic activity [2]. During the first eight weeks, the alveolar bone is replaced by reticular bone and subsequently by mature lamellar bone, with a marked loss of the buccal wall [1,3]. Barrier membranes play a key role in ridge preservation by excluding soft tissue invasion, maintaining regenerative space, stabilizing graft materials, and protecting the blood clot during early healing. These mechanisms are essential for predictable bone regeneration. When performed in combination with grafts and barrier membranes, alveolar preservation has shown good results [4].

Most ridge remodeling occurs during the early healing phase following tooth extraction. Clinical and radiographic studies have reported substantial horizontal and vertical dimensional changes during the first year after extraction. However, the magnitude of these alterations varies considerably depending on the anatomic site (maxilla versus mandible), buccal plate thickness, socket wall phenotype, baseline defect severity, and the measurement methodology used in the studied populations [4,5]. Tomographic evaluations of alveolar ridge preservation procedures have also demonstrated variability in dimensional outcomes depending on the grafting protocol and measurement levels employed, further underscoring that reported dimensional changes should be interpreted within the specific clinical and methodological context of each study [5]. These decreases compromise the emergence profile and increase the difficulty of implant-based prosthetic rehabilitation, particularly in patients with multiple extractions or stage IV periodontitis.

From the perspective of implantology, the residual bone volume determines the number, diameter, length, and distribution of implants, as well as the possibility of achieving adequate primary stability while maintaining critical anatomical structures [6]. In cases of severely reabsorbed ridges, additional regenerative procedures (e.g., guided bone regeneration, block grafts, or maxillary sinus elevation) are often required. However, the indication depends on site-specific anatomy, defect morphology, and prosthetic treatment objectives. These procedures may be associated with increased longer treatment time and higher costs, but they are associated with increased morbidity. Therefore, alveolar preservation has been proposed as a preventive strategy for teeth with bleak outcomes, with the aim of maintaining bone and soft tissue volume and optimizing recipient sites for future implant placement [4,7].

When performed in combination with grafts and barrier membranes, alveolar preservation has shown good results [4,8]. Compared with non-resorbable membranes, resorbable collagen membranes avoid the need for a second surgical procedure for membrane removal and are associated with favorable soft tissue healing. Their degradation profile and biological properties contribute to predictable clinical performance in guided bone regeneration procedures [8-10].

The clinical performance of collagen membranes depends on properties such as thickness, mechanical stability, and barrier duration. Membranes providing greater structural stability may be preferable in defects requiring space maintenance, whereas faster-resorbing membranes may be suitable for contained extraction sockets [10,11]. Bone graft materials support regeneration through distinct mechanisms. Autografts provide osteogenic, osteoinductive, and osteoconductive properties, whereas xenografts primarily act as osteoconductive scaffolds that facilitate host-mediated bone formation and maintain defect volume during healing [12].

Although autografts are considered the gold standard, their rapid remodeling may reduce long-term volume stability. Combining autografts with xenografts has been proposed to improve structural maintenance compared with autograft alone, particularly in defects requiring enhanced space preservation. However, they remain dependent on defect characteristics and surgical protocol [13].

Several studies have used CBCT to evaluate dimensional changes of the alveolar ridge, including horizontal width at different subcrestal levels (1-5 mm) and vertical changes at buccal and lingual aspects following ridge preservation procedures using bovine xenografts and collagen membranes [5,6,14]. However, standardized long-term evaluations in complex cases such as Stage IV periodontitis with multiple extractions remain limited. The objective of this clinical case was to use CBCT to evaluate the multidimensional changes in the ridge after preservation procedures with a resorbable collagen membrane from the porcine pericardium and a bovine bone xenograft, as well as to document the feasibility of the subsequent placement of multiple implants and to describe the clinical outcome in this patient, in whom implant placement and prosthetic rehabilitation were achieved without the need for additional regenerative procedures.

## Case presentation

A 61-year-old male nonsmoker with no relevant systemic history visited the Periodontology and Implantology Clinic for severe dental mobility and rehabilitation with dental implants. Clinical examination revealed grade III mobility of teeth 11, 12, 13, 15, 16, 21, 22, 23, 28, 33, 34, 43, 44, and 48, with generalized periodontal attachment loss.

Baseline periodontal assessment confirmed Stage IV periodontitis, with generalized probing depths of 6-9 mm, bleeding on probing in over 50% of sites, advanced attachment loss, and radiographic bone loss exceeding 50% of root length, including vertical defects and furcation involvement. Teeth with Grade II-III mobility and unfavorable crown-to-root ratios were classified as hopeless. An interdisciplinary treatment plan was developed with the prosthodontics department, and initial non-surgical periodontal therapy was implemented prior to surgery. A staged approach alongside preservation followed by delayed implant placement was chosen to optimize the conditions of the recipient site, allowing for resolution of inflammation, infection control, and stabilization of hard and soft tissues after extractions. This approach promotes the preservation of horizontal and vertical bone volume, improves the quality of the bone bed, and increases the predictability of osseointegration, especially in patients with advanced periodontitis, where immediate placement could compromise primary stability and long-term prognosis.

Initial tomographic evaluation

**Figure 1:**
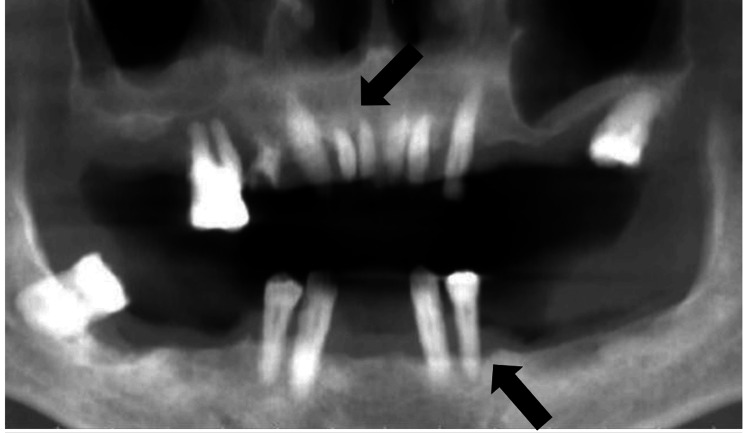
Preoperative cone-beam computed tomography image. The arrows indicate bone loss in both the maxilla and mandible.

After periodontal diagnosis and completion of the initial nonsurgical phase, preoperative CBCT was performed, which confirmed advanced bone loss in the maxilla and mandible (Figure 1).

Given the unfavorable results of the affected dental elements, extractions of all teeth were planned with preservation of the alveolar ridge at the sites designated for future implant rehabilitation.

**Figure 2:**
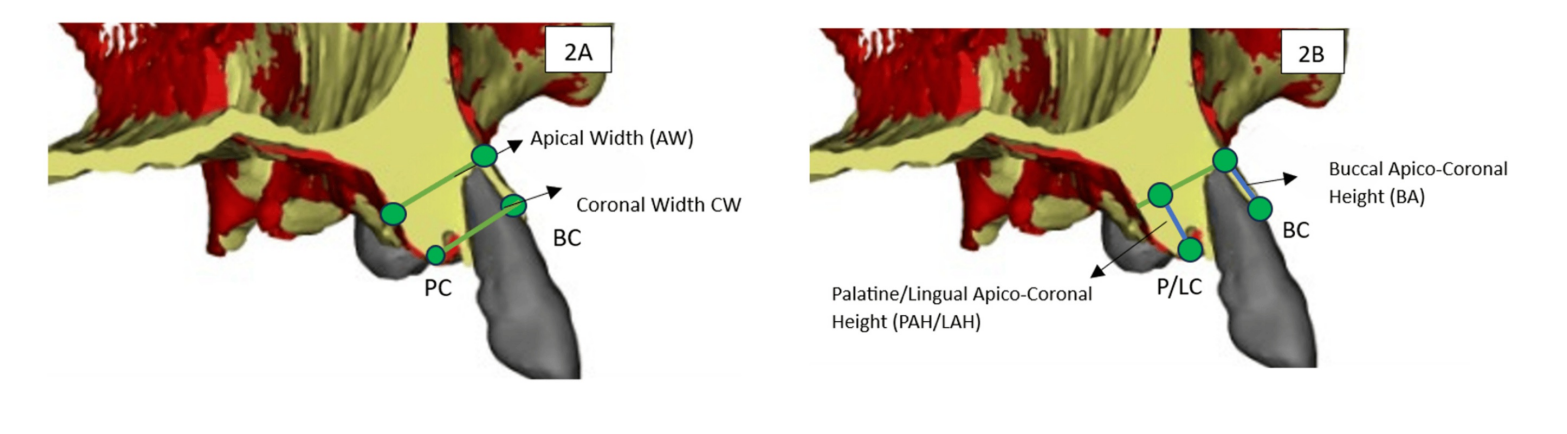
(A) Diagram of the measurements of the alveolar ridge width. (B) Diagram of the measurements of the alveolar ridge height. The figure shows 3D slices from the preoperative CT scan, obtained using Slicer 5.8.1 software (International open-source community driven by Brigham and Women’s Hospital and the MIT AI Lab, USA). BR: buccal ridge; P/LR: palatal/lingual ridge; BA: buccal apico-coronal height; PAC/LAC: palatal/lingual apico-coronal height; CW: coronal width

The following four parameters were defined for the dimensional analysis (Figure 2): coronal width (CW), the horizontal distance between the vestibular and palatal/lingual bone crests at the coronal level; apical width (AW), the horizontal distance at the apical level; buccal apicoronal height (BH/AC), the distance from the apex to the buccal ridge; and palatal/lingual apicoronal height (PLH/PAC-LAC), the distance from the apex to the palatal or lingual crest.

Baseline CBCT measurements were obtained from all extraction sockets included in the preservation protocol in each arch. In the maxilla, [#23,#22,#21,#11,#12,#13], and in the mandible, [#33,34,44,45], were analyzed. For each socket, ridge width was measured at predefined coronal and apical levels, and vertical dimensions were assessed at the buccal and palatal/lingual plates.

The reported values represent the mean measurements calculated across all preserved sites within each arch for longitudinal comparison. All measurements were performed by a single calibrated operator following a standardized CBCT assessment protocol.

Maxillary surgical procedure

**Figure 3:**
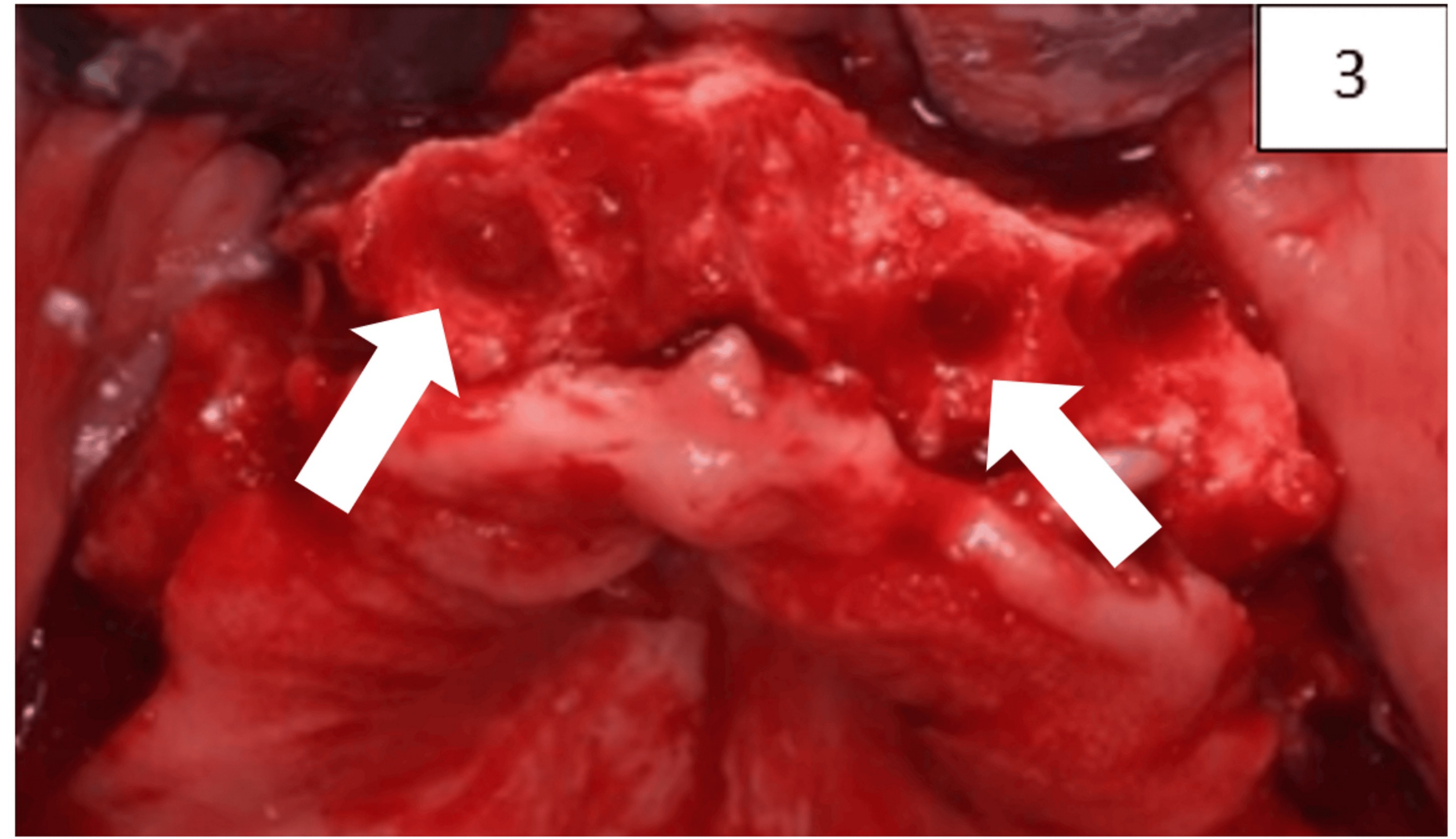
Atraumatic extraction. The arrows indicate extraction without bone trauma.

In the first phase, teeth 11, 12, 13, 15, 16, 21, 22, and 23 were atraumatically extracted (Figure 3) under local anesthesia. An autograft was harvested from the adjacent surgical area using a bone scraper (Supertack, MC Bio, Lomazzo, Italy) and mixed in an approximate 30/70 volume ratio with a previously hydrated bovine bone xenograft (InterOss®, SigmaGraft, CA, USA). The autogenous component was added to provide osteogenic and osteoinductive potential, while the xenograft acted primarily as an osteoconductive scaffold to enhance volumetric stability of the grafted socket.

**Figure 4:**
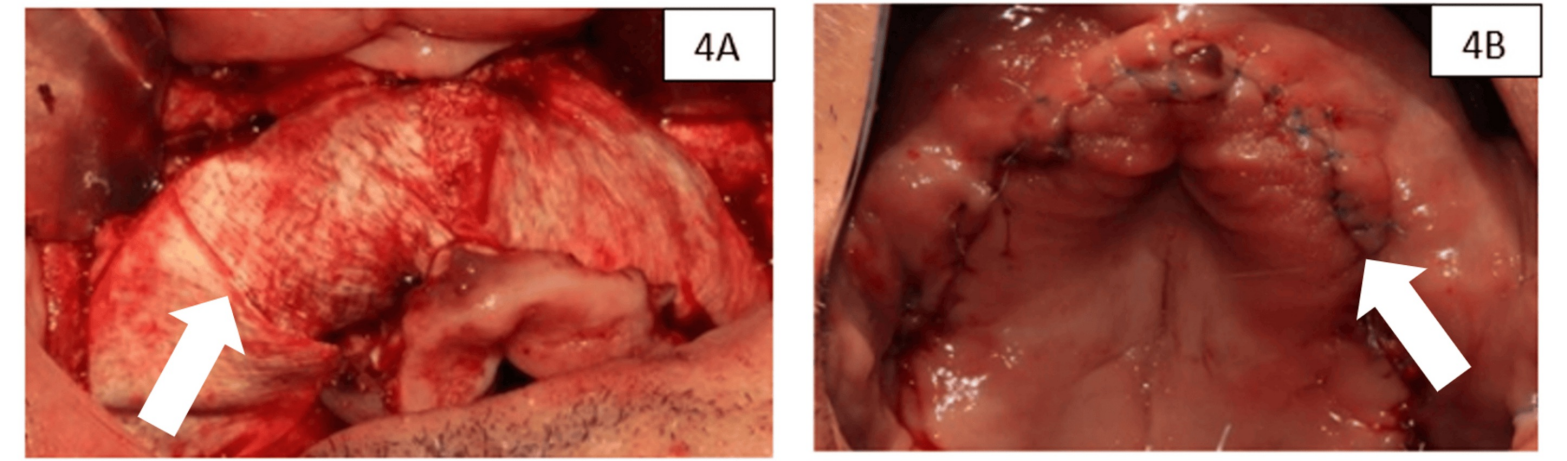
(A) Stabilization of the membrane with tacks. (B) Sutured flap. The arrow indicates the suture points.

A resorbable native porcine pericardial collagen membrane (InterCollagen® Guide Membrane, SigmaGraft, CA, USA) was adapted and stabilized on the palatal surface. The alveoli were subsequently filled with the autologous graft-xenograft mixture, and the membrane was fixed on the buccal side with tacks (Supertack, MC Bio, Lomazzo, Italy) (Figure 4). Finally, the mucoperiosteal flap was repositioned and sutured with single stitches and horizontal mattress sutures with 6-0 nylon monofilament (RESOLON®, RESORBA®, Nuremberg, Germany) (Figure 4).

Mandibular surgical procedure

**Figure 5:**
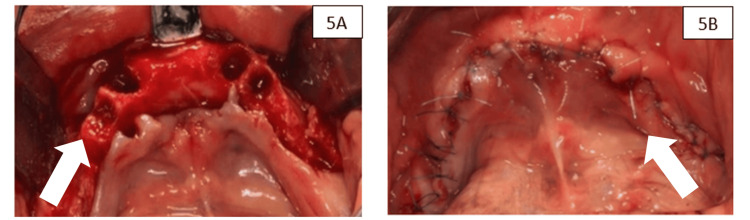
(A) Atraumatic extraction. The arrow indicates extraction without bone trauma. (B) Sutured flap. The arrow indicates the suture points.

In the second phase, atraumatic extractions of teeth 33, 34, 43, and 44 were performed (Figure 5). Tooth 48 was extracted, but not at the same stage as teeth 33, 34, 44, and 43; it was extracted earlier, and no alveolar preservation was performed. At these sites, a hydrated bovine xenograft (InterOss®, SigmaGraft, CA, USA) was placed and closed with single stitches and horizontal mattress sutures (Figure 5). The preservation protocol differed between arches due to site-specific anatomical and defect characteristics. In the maxilla, the presence of thinner buccal plates and a higher risk of horizontal collapse justified the use of a combined autogenous bone-xenograft mixture covered with a porcine pericardium collagen membrane. In contrast, the mandibular extraction sockets exhibited thicker cortical plates and intact socket walls, allowing stabilization of the hydrated xenograft without membrane coverage. Therefore, the interventions were tailored to the local clinical conditions and were not intended for direct inter-arch comparison.

At the end of both phases, an immediate provisional prosthesis was placed to maintain function and guarantee the stability of the soft tissues.

Postoperative management and evolution of soft tissues

**Figure 6:**
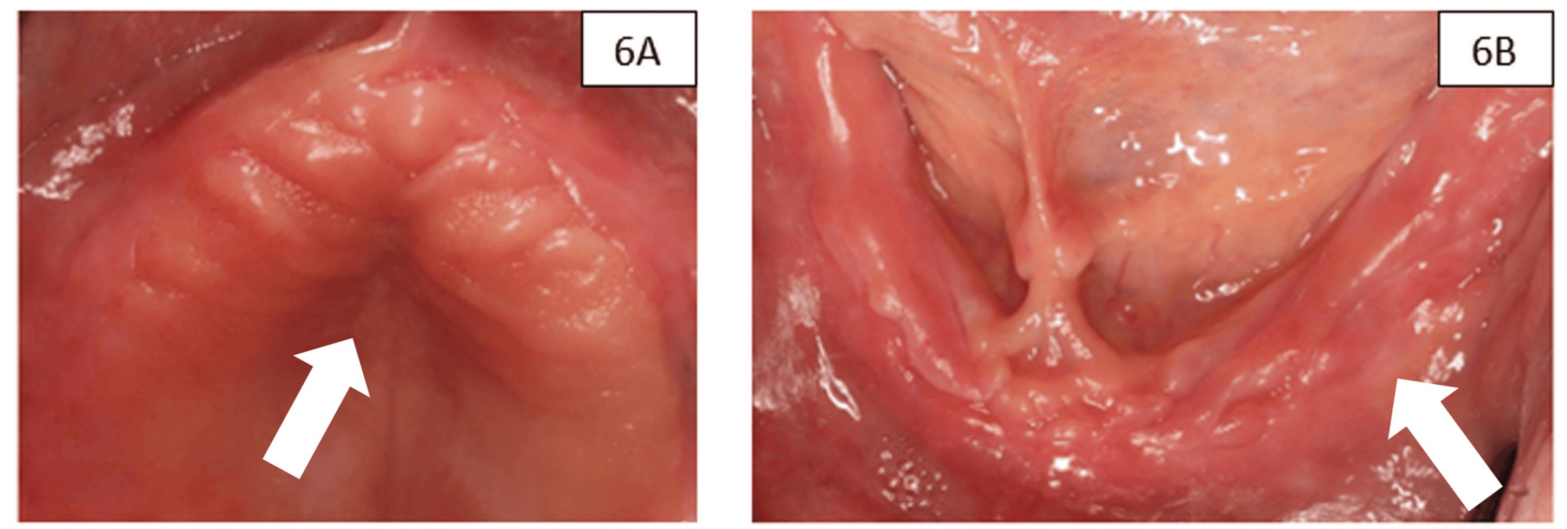
(A) Postsurgical image of maxilla. (B) of the mandible. The arrows indicate keratinized tissue

Postoperative management included ibuprofen 600 mg every 8 hours for five days and amoxicillin 500 mg every 8 hours for seven days. Patients were instructed to rinse with 0.12% chlorhexidine gluconate twice daily for 14 days. Sutures were removed at 15 days, and at 30 days, adequate healing was observed, with thick and stable keratinized tissue covering the preserved sites in both the maxilla and mandible (Figure 6). No major postoperative complications, such as dehiscence, membrane exposure, or infection, were observed.

Tomographic control at 12 months and the peri-implant phase

**Figure 7:**
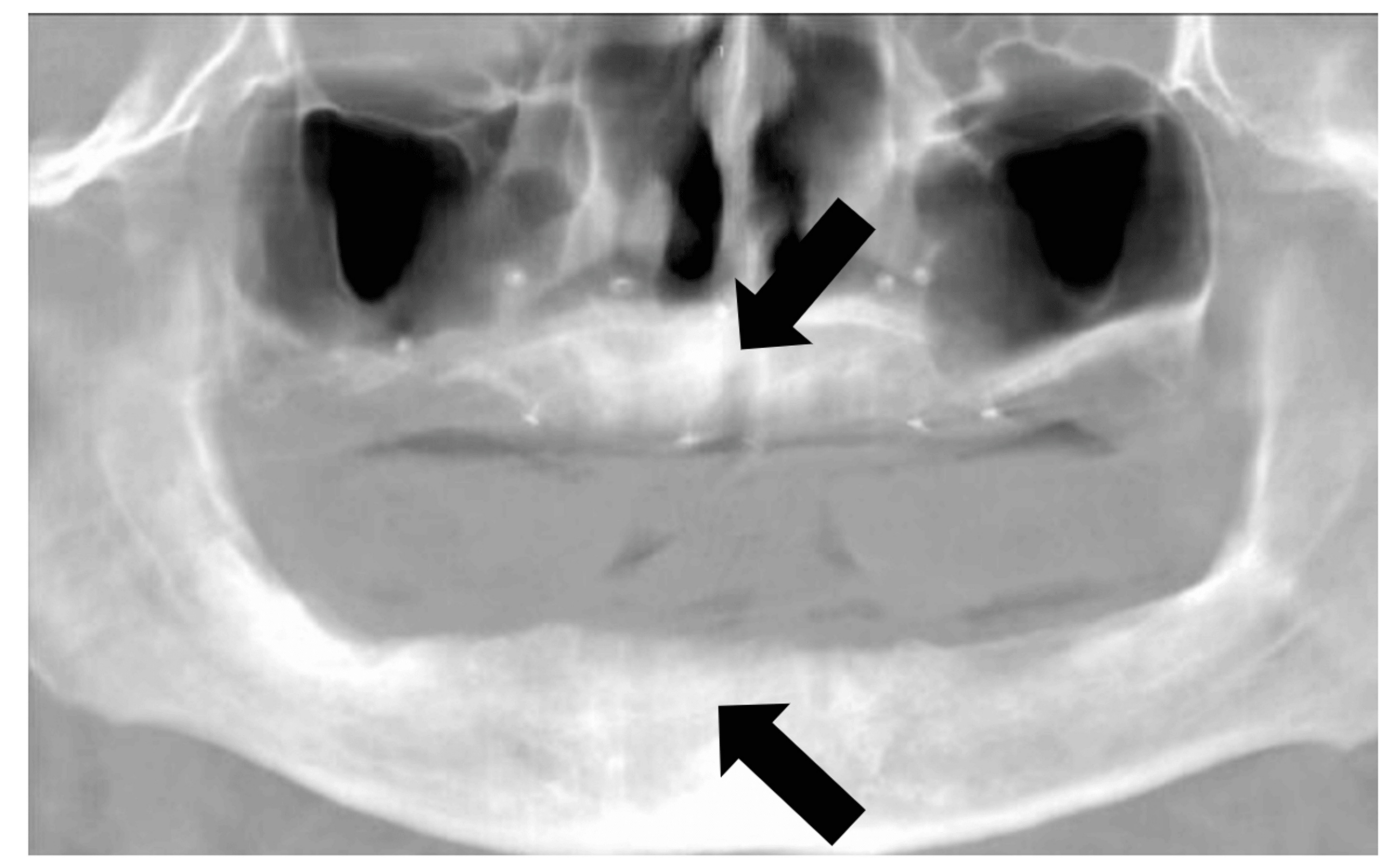
Tomographic image at 12 months. The arrows indicate areas of dimensional stability and minimal change as confirmed by standardized CBCT measurements and image superimposition.

At 12 months, a new CBCT scan was obtained (Figure 7), and dimensional measurements were repeated at the same predefined reference points. For longitudinal analysis, baseline and follow-up tomographic datasets were superimposed using 3D Slicer software (version 5.8.0) through rigid registration based on stable craniofacial anatomical landmarks, including the cranial base, anterior nasal spine, and orbital floor. Alignment accuracy was verified visually in axial, sagittal, and coronal planes prior to measurement to ensure consistency in voxel correspondence and spatial orientation.

**Figure 8:**
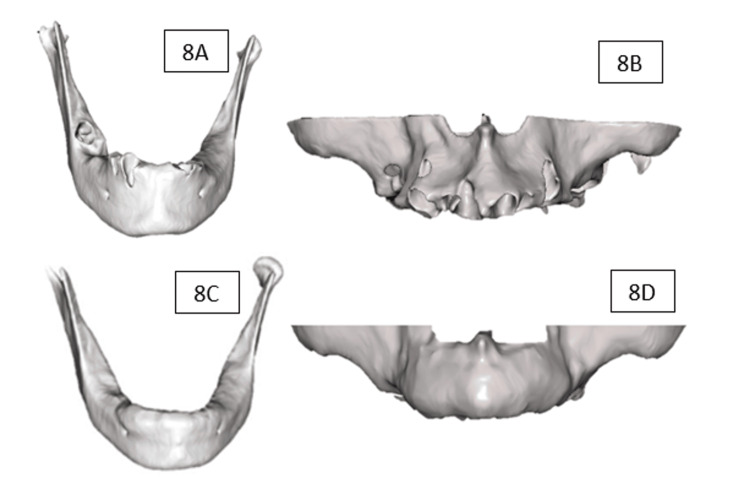
(A) Mandible at baseline prior to tooth extraction. (B) Maxilla at baseline prior to tooth extraction. (C) Mandible at 12-month follow-up. (D) Maxilla at 12-month follow-up. The figure shows 3D slices from the preoperative CT scan and at 12 months, obtained using Blue Sky Plan 4 software (Blue Sky Bio, LLC, Libertyville, IL, USA)

**Figure 9:**
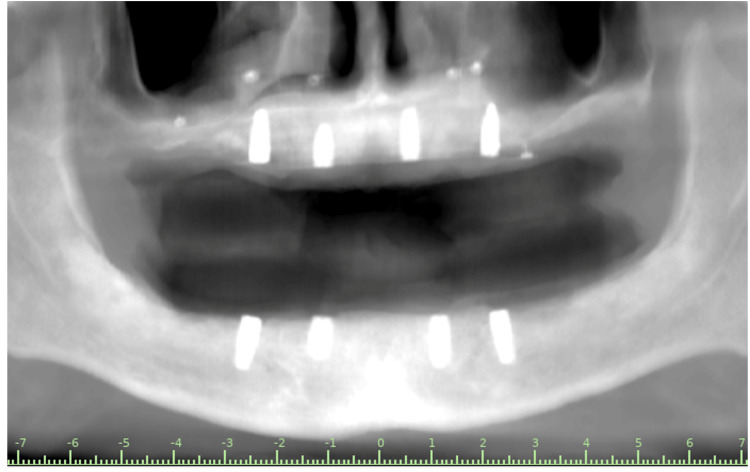
Tomographic image at 3 months

The overall multidimensional stability and focal horizontal gains allowed for implant phase planning without the need for additional bone regeneration (Figure 8). In accordance with the prosthetic plan, four implants were placed in the maxilla (12, 15, 22, and 25) and four were placed in the mandible (43, 45, 33, and 35). In accordance with the prosthetic plan, four implants were placed in the maxilla (12, 15, 22, and 25) and four in the mandible (43, 45, 33, and 35). The insertion torque values ​​were 45 N·cm in the maxilla and mandible, indicating adequate primary stability at the time of placement. Three months after implant placement, a control CBCT scan was obtained. The dimensional differences observed between measurements ranged from 0.2 to 0.6 mm across the evaluated sites. These variations fall within the expected range of CBCT measurement variability and rigid registration error, supporting the interpretation of early dimensional stability at the recipient sites (Figure 9). This allowed us to continue with definitive prosthetic rehabilitation through implant-supported overdentures, with satisfactory functional and aesthetic performance at the three-month clinical follow-up.

Results

The baseline tomographic measurements and those obtained at 12 months showed overall dimensional preservation of the ridge in both jaws, with slight horizontal gains and minimal vertical losses. In the maxilla, the average CW increased from 8.2 to 8.4 mm, and the AW increased from 8.8 to 9.9 mm; in the mandible, the CW increased from 9.2 to 9.6 mm, and the AW increased from 9.9 to 10.1 mm. Vertically, the buccal height decreased from 5.8 to 5.2 mm in the maxilla and from 4.2 to 3.6 mm in the mandible, whereas the PL height decreased from 6.0 to 5.8 mm in the maxilla and from 4.6 to 4.4 mm in the mandible (Table 1). For all vertical parameters, the loss was less than 1 mm.

**Table 1:** Dimensional averages of the alveolar ridge: baseline vs. 12 months (mm). Average dimensional values obtained by CBCT at baseline and 12 months at preserved sites. CW: coronal width; AW: apical width; BH/BAC: buccal height; PLH/PAC-LAC: palatal/lingual apicoronal height

Arch	Baseline CW	12-month CW	Baseline AW	12-month AW	Baseline BH	12-month BH	Baseline PLH	12-month PLH
Maxillary	8.2	8.4	8.8	9.9	5.8	5.2	6.0	5.8
Mandibular	9.2	9.6	9.9	10.1	4.2	3.6	4.6	4.4

To describe the dimensional behavior in greater detail, the reabsorption and average gain per parameter were analyzed (Table 2). In this table, the average resorption corresponds to the average decrease at the sites that presented dimensional loss, whereas the average gain reflects the average increase at the sites that showed an increase with respect to the baseline values. In general, reabsorption was concentrated in the vertical buccal component height, whereas the greatest gains were observed in the horizontal parameters, namely, the maxillary and mandibular AW (Table 2).

**Table 2:** Resorption and average gain according to clinical parameters (mm). Average changes at 12 months with respect to baseline values. CW: coronal width; AW: apical width; BH: buccal height; PLH: palatal height

Parameter	Arch	Average reabsorption	Average gain
CW	Maxillary	0.62	0.96
CW	Mandibular	0.83	1.44
AW	Maxillary	0.66	2.77
AW	Mandibular	0.41	0.77
BH	Maxillary	0.80	0.10
BH	Mandibular	0.61	0.00
PLH	Maxillary	0.62	0.35
PLH	Mandibular	0.33	0.07

The volume stability observed after 12 months allowed the planning of the placement of eight implants without the need for additional regenerative procedures. At the recipient sites, the pre-implant and 3-month post-placement CBCT evaluation data showed minimal variations in all parameters, which was compatible with the favorable consolidation of the preserved bed (Tables 3, 4).

**Table 3:** Dimensional averages implant recipient sites: implant vs. 3 months (mm). CW: coronal width; AW: apical width; BH: buccal height; PLH: palatal height

Arch	Baseline CW	3-month CW	Baseline AW	3-month AW	Baseline BH	3-month BH	Baseline PLH	3-month PLH
Maxillary	10.71	10.30	13.08	13.08	9.02	9.01	8.37	8.35
Mandibular	9.62	9.18	9.83	9.83	9.27	9.24	9.94	9.91

**Table 4:** Average changes at the implant recipient sites: baseline vs. 3 months (mm). Average changes between pre-implant and 3-month CBCT values; values close to zero reflect volumetric stability. CW: coronal width; AW: apical width; BH: buccal height; PLH: palatal height

Parameter	Arch	Average change (mm)
CW	Maxillary	0.41
CW	Mandibular	0.45
AW	Maxillary	0.00
AW	Mandibular	0.00
BH	Maxillary	0.02
BH	Mandibular	0.03
PLH	Maxillary	0.02
PLH	Mandibular	0.03

Taken together, these results indicate that alveolar preservation with a porcine pericardium membrane and bovine xenograft resulted in maintenance or slight improvement of horizontal ridge dimensions and limited vertical resorption (<1 mm). At follow-up, all implants remained clinically stable, with no mobility, pain, or peri-implant complications, and tomographic evaluation showed stable crestal bone levels, supporting early implant stability.

## Discussion

The findings from this clinical case suggest that after the alveolar ridge was preserved following multiple extractions in a patient with stage IV periodontitis, bone remodeling presented a heterogeneous pattern, with some areas of limited reabsorption and others with localized dimensional gains. The greatest loss was concentrated in the vertical vestibular components of both the maxilla (0.80 mm) and mandible (0.61 mm) (Table 2), which is consistent with the findings of Araujo and Lindhe [2], who described a greater vulnerability of the vestibular plate because of its relatively low thickness and dependence on the periodontal vasculature.

Overall, the average horizontal reduction was approximately 0.63 mm, and the vertical reduction was 0.56 mm; these results are consistent with those reported in systematic reviews, such as that of Barootchi et al. [15]. However, in this clinical case, relevant punctual gains were observed, particularly in the maxillary AA (2.77 mm) and mandibular AC (1.44 mm), which were most likely due to the synergistic effects of the bovine xenograft, local autologous graft, and mechanical stability provided by the porcine pericardial membrane.

Clinical reports and reviews by Barootchi [15], Cardaropoli [16], and Starch-Jensen [17] have shown that osteoconductive biomaterials used for guided bone regeneration favor the formation of new bone and decrease crestal collapse. Likewise, clinical trials have shown that sealing the alveolus with barrier membranes contributes to preserving the crest width, particularly when stable tissue closure is achieved. The results of this case are consistent with this evidence, showing that the combination of a xenograft and membrane maintained a horizontal dimension with a vertical loss of less than 1 mm.

From a therapeutic perspective, it is clinically relevant that no site required additional grafts during the implantation phase. The obtained volumetric stability allowed the placement of eight implants with adequate primary stability, thereby simplifying implant-supported rehabilitation.

This behavior is consistent with that reported by other studies, according to which alveolar preservation decreases the need for subsequent regenerative procedures and optimizes the therapeutic route for more predictable rehabilitation [18].

Although specific evidence on collagen membranes derived from the porcine pericardium is still limited compared with that available for other porcine collagen membranes, the dimensional results and control of vestibular resorption and implant viability without additional regeneration observed in this case suggest a clinical performance comparable to that of the most studied alternatives. Recent studies combining bovine xenografts with porcine membranes have documented acceptable vertical stability and a reduced need for additional procedures, even in the maxillary posterior regions, where sinus pneumatization is a frequent challenge. This case report has inherent limitations, including its single-patient design and the absence of a control group. Dimensional changes were assessed using CBCT, which may be subject to methodological variability related to measurement and image registration. In addition, no histological evaluation was performed to confirm true bone regeneration. Therefore, the findings should be interpreted as demonstrating clinical feasibility in this patient rather than establishing generalizable treatment predictability [19].

Finally, future studies with case series or comparative designs are needed, as well as long-term follow-ups focused on peri-implant bone stability and prosthetic results, which are necessary to validate the reproducibility of these findings.

## Conclusions

In this clinical case, alveolar ridge preservation using a resorbable porcine pericardial collagen membrane and bovine xenograft was associated with favorable dimensional stability following multiple extractions. Vestibular resorption remained below 1 mm, and the horizontal ridge dimensions remained stable with minimal changes over time. This dimensional stability allowed the planning and placement of eight implants without additional regenerative procedures, facilitating implant-supported rehabilitation in this patient with stage IV periodontitis.

This case suggests that the combination of a porcine pericardial membrane and bovine xenograft may represent a feasible pre-implant preventive strategy in patients with advanced periodontal loss requiring extensive rehabilitation. However, additional well-planned clinical studies are needed to confirm these findings and determine whether this treatment approach and its outcomes can be generalized to broader patients.
